# High Precision Low-Speed Control for Permanent Magnet Synchronous Motor

**DOI:** 10.3390/s20051526

**Published:** 2020-03-10

**Authors:** Xianqi Xia, Bao Zhang, Xiantao Li

**Affiliations:** 1 Changchun Institute of Optics, Fine Mechanics and Physics, Chinese Academy of Sciences, Changchun 130033, China; xiaxianqi16@mails.ucas.edu.cn (X.X.); lixiantao@ciomp.ac.cn (X.L.); 2 University of Chinese Academy of Sciences, No.19, Yuquan Rd., Beijing 100049, China

**Keywords:** permanent magnet synchronous motor, inertia stability, sliding mode control, iterative learning control, extended state observer

## Abstract

Due to the process defects and imperfection of drivers, permanent magnet synchronous motors (PMSM) are problematic to control. There is still a lack of effective high-performance control methods for inertial stabilized platforms based on PMSM currently. At present, the most frequently used method is sliding mode control (SMC), but traditional sliding mode control cannot overcome the contradiction between high performance and system chattering. In order to solve this problem and improve the system reliability and pointing accuracy, a new approach law for the sliding mode controller is proposed in this paper. In view of the large periodic torque ripple in PMSM, an iterative learning controller (ILC) is introduced to compensate for the disturbance. Based on these, aimed at suppressing all kinds of real-time disturbances in the working environment of the system, the extended state observer (ESO) is brought into the servo system to observe the lumped disturbance of the system, and the total disturbance observed is compensated into the sliding mode controller, so as to better suppress the system chattering and enhance the system’s ability of resisting external disturbance. Experiments are carried out on an inertial stabilization platform based on DSP + CPLD. The final experiments verify that the SMC with the new approach, combined with ILC and ESO, is of outstanding performance when compared with the traditional proportional integral (PI) + disturbance observer (DOB) control scheme.

## 1. Introduction

At present, a direct current (DC) torque motor is widely used to drive the load in the inertial stabilization platform. However, the DC torque motor has the shortcomings of large volume and poor heat dissipation, as well as the high-frequency and non-linear interference brought to the system by using mechanical commutation, which reduces the system’s reliability. Owing to the advantages of high power density, high air gap flux density and small volume, permanent magnet synchronous motors (PMSM) become the first choice for replacing the DC torque motor [1]. Nevertheless, due to the defects of the PMSM itself and the poor working environment of various inertial stabilization platforms, it is arduous for linear control schemes, such as proportional integral (PI) control and the linear quadratic regulator, to achieve desirable control performance. For the inertial stabilization platform for long-distance reconnaissance in particular, the servo stabilization accuracy directly affects the imaging quality [2]. Accordingly, a more advanced control scheme needs to be introduced into the servo system of the inertial stabilization platform driven by PMSM.

For the influence of various interferences on the performance of PMSM speed control system, [3,4,5] make a detailed analysis. In view of the cogging effect caused by the PMSM manufacturing process, [6,7,8] use iterative learning method to suppress the periodic disturbance of mechanical position, which obtain certain effect. For the influence of current detection error on the system, a measurement error observer is proposed in [9] for real-time observation and compensation. According to the dead time effect of the inverter, [10,11] provide a detailed analysis and compensation scheme.

All of the above targeted control methods achieve satisfactory results. Nonetheless, the control system will be too complex if we design separate controllers for every kind of disturbance. For the current loop whose working frequency is more than 4 kHz in particular, the calculation may not be able to complete in one single cycle if the program is too large.

In the low-speed system, because the output torque of the motor is small, and the cogging torque is the same order of magnitude or even bigger, the performance of the system is greatly affected by the cogging torque. An iterative learning controller (ILC) is widely used in PMSM to overcome the periodic cogging torque disturbance because of its excellent suppression effect on periodic disturbance and easy to realize in digital system [6,7,8]. In reference [12], an open-loop ILC controller is designed, which makes the design of ILC simpler, but it still has the problem of slow convergence. Reference [13] designs an ILC based on the frequency domain, which can suppress periodic disturbance of several frequencies at the same time. However, it is difficult to combine with other controllers in the system. In our experimental system, the main periodic disturbance is only cogging torque, so there is no need to design a controller for other frequency periodic disturbances.

In the past decade, sliding mode control has become the focus of alternating current (AC) servo drive control research for its fast response, strong robustness to external disturbances and insensitivity to model uncertainty. Sliding mode control can be divided into two stages: approach stage and sliding stage. Before the system state reaches the sliding surface (approach phase), the controller outputs the control variable which makes the system state approach to the sliding surface. When the system state reaches the sliding surface, the system enters the sliding phase. Once the state of the system is on the sliding hyperplane, the state of each variable of the system is determined by the defined sliding surface, and will not be affected by external disturbance and system uncertainty. These advantages of sliding mode control have been widely used in the position and speed control system of the permanent magnet synchronous motor [14,15,16,17].

In the basic sliding mode control, the upper bound of the system disturbance must be determined for the designing of controller parameters to ensure the stability of the system. However, for most engineering applications, the size of the disturbance cannot be obtained in advance. Therefore, the upper bound of the external disturbance is usually set to a large value to design the controller to meet the reachability condition of the sliding mode controller. It is disappointing that the controller parameters designed by this method are too conservative to achieve desirable performance. At the same time, in order to make the system state reach the sliding surface quickly to achieve better control effect, the controller parameter k must take a larger value, but the increase of the parameter will make the system state across the sliding surface more frequently. Consequently, the system output contains a large number of high-frequency components, which will cause the system to vibrate violently and even make it unstable. In the traditional sliding mode controller without disturbance compensation, the parameter k must be large enough to meet the sliding mode reachability condition, resulting inevitably in system chatter. But the chattering of the system will seriously affect the imaging quality and system stability, even bring serious irreversible physical damage to the platform.

The extended state observer (ESO) is widely used to observe system disturbances. Compared with the traditional disturbance observer (DOB), ESO as a “closed-loop observer” that has faster convergence speed and smaller phase lag. The advantages of ESO are analyzed in detail in reference [18]. In recent years, the compound control scheme of extended state observer and various controllers has been applied to the PMSM control system. Reference [19] combines predictive functional control with ESO for PMSM speed control. In reference [20], ESO is applied to the current loop of PMSM, and a current loop controller with strong robustness and high bandwidth is designed in combination with predictive control. The above ESO applications have achieved good results

Based on the above background, a PI type iterative learning controller is designed to iterate out the equivalent control variable of the cogging torque and compensate the control variable into the system model. A sliding mode controller with a new approach law is designed to reduce the chattering caused by the discontinuous switching of control variables. At the same time, the disturbance compensation term is introduced into the sliding mode controller. The ESO is brought in to observe the total disturbance of the system in real time, and the observed disturbance is employed to the sliding mode controller, so that the reaching law still satisfies the reachability condition at a relatively small value, which improves the performance of the system, as well as reducing the chattering of the system further.

In this paper, the control method of PI + DOB is compared with the proposed ILC + sliding mode control (SMC) + ESO. The turntable driven by PMSM is used as the experimental equipment. Simulation and experimental results show the superiority of the proposed scheme.

## 2. Mathematical Model of Permanent Magnet Synchronous Motors (PMSM)

We consider a surface-mounted PMSM. Suppose that: (1) the magnetic flux of the motor is not saturated, (2) the eddy currents and hysteresis losses are negligible, (3) the three-phase stator windings of PMSM are sinusoidally distributed in space. For the purpose of control design, $\left(i_{d},\;i_{q},\;\omega \right)$ are chosen as state variables. The mathematical model of PMSM can be expressed as follows [21]:

(1) $$\left(\begin{array}{c} {\dot{i}}_{d}\\ {\dot{i}}_{q}\\ \dot{\omega } \end{array}\right)=\left(\begin{array}{ccc} -R/L & p\omega & 0\\ -p\omega & -R/L & p\psi_{f}/L\\ 0 & K_{t}/J & -B/J \end{array}\right)\left(\begin{array}{c} i_{d}\\ i_{q}\\ \omega \end{array}\right)+\left(\begin{array}{c} u_{d}/L\\ u_{q}/L\\ -T_{d}/J \end{array}\right)\;$$

where

$i_{d},\;i_{q}$ stator currents along the d and q axes
$u_{d},\;u_{q}$ stator voltages along the d and q axes
p number of pole pairs
R stator resistance
L stator inductance
$\psi_{f}$ Rotor Flux
$K_{t}$ torque coefficient
ω mechanical angular speed
B frictional coefficient
J inertia
$T_{d}$ disturbance torque (including cogging torque $T_{cog}$ and other real-time disturbance)

In our experimental system, the phase current is converted into voltage through the acquisition and amplification circuit, and then $I_{d},\;I_{q}$ can be solved in reverse after ADC sampling. The speed ω is measured by micromachined gyroscope. Stator resistance R, inductance L, and pole pair p are known parameters of the motor. $\psi_{f},\;K_{t},\;B,\;J,\;T_{d}$ are unknown parameters.

In order to ensure the stability of the controller and observer designed based on this model, the current of the system should not be too large, so as to avoid exceeding the linear working range of the amplifier circuit. The angular velocity shall be within the measurement range of the gyroscope.

## 3. Design of Iterative Learning Controller (ILC)

### 3.1. Use ILC to Restrain Cogging Torque

The core idea of ILC is to correct the output of the current controller by using the last output of the controller and the current error, so as to achieve the purpose of suppressing periodic disturbance.

The ILC can be divided into two types according to the error information it uses: PCF type (using the tracking error of the last iteration) and CCF type (using the tracking error of the current iteration). According to the specific algorithm of error information, ILC can be divided into P type, PI type and PID type. [22]

**Figure 1:**
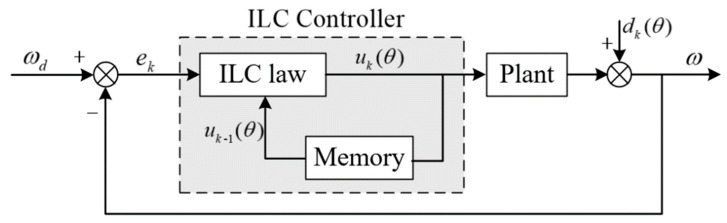
Block diagram of iterative learning controller (ILC).

The CCF type PI iterative learning controller is used in this paper. Since the magnitude of cogging torque is only related to the angular position, the ILC in the space domain is used. The block diagram of the control scheme is as in Figure 1:

We use the subscript k to represent the number of iterations, θ to represent the current mechanical angle, $\omega_{d}$ to represent the given speed, ω to represent the actual speed, u to represent the output of ILC controller, and d to represent the disturbance torque.

PI type control is adopted in the ILC:

(2) $$u_{k}(\theta )=u_{k-1}(\theta )+F\left(e_{k}(\theta )\right)$$

(3) $$F\left(e_{k}(\theta )\right)=G_{P}e_{k}(\theta )+G_{I}\int e_{k}(\theta )dt$$

the system output can be computed as follow according to Figure 1:

(4) $$\omega_{k}=Pu_{k}+d_{k}$$

where P is the system transfer function.

Combining Equations (2)–(4), we can easily get the tracking error of the $k^{th}$ iteration:

(5) $$e_{k}=e_{k-1}-P\left(G_{P}e_{k}+G_{I}\int e_{k}dt\right)+d_{k-1}-d_{k}$$

If the disturbance d is a periodic function of position, that is $d_{k}(\theta )=d_{k-1}(\theta )$. Then the expression of tracking error in the above equation becomes:

(6) $$e_{k}=e_{k-1}-P\left(G_{P}e_{k}+G_{I}\int e_{k}dt\right)$$

It can be seen that the tracking error of the system is independent of the disturbance, and the external disturbance is completely suppressed.

The actual operation of the system is affected by various disturbances, such as current sampling error, inverter dead time effect, and shaft friction, etc. For our experimental system, due to the high performance of the amplifier circuit and ADC, the short dead time of the inverter, and the low-speed operation of the system, the current sampling error and “dead time effect” have little impact on the system. Therefore the disturbance comes mainly from the cogging effect caused by the manufacturing process defects of the motor and the friction force of the shaft system. when the system is in the speed reversal, the friction torque makes a big impact. If the motor is running in a single direction, according to the Coulomb model, the friction of the shafting can be considered as a constant value. When the motor runs at a constant speed, the system torque is balanced, that is, the output torque of the motor is completely used to overcome the cogging torque and friction torque. That is, the following equation holds:

(7) $$\begin{array}{l} if\;\omega =\mathrm{constant}\text{:}\\ \left\{ \begin{array}{l} Ku_{+}(\theta )=T_{c}(\theta )+T_{f}\\ Ku_{-}(\theta )=T_{c}(\theta )-T_{f} \end{array}\right. \end{array}$$

where, $u_{+}(\theta )$ is the control variable inputted into the system when the motor is at the angle θ and ω>0, K is the ratio coefficient (constant) between the control variable and the torque, $T_{c}(\theta )$ is the cogging torque at the angle θ, and $T_{f}$ is the friction torque (the positive direction is specified to be consistent with the negative direction of the speed).

From Equation (7), it is easy to obtain:

(8) $$T_{c}(\theta )=\frac{K\left(u_{+}(\theta )+u_{-}(\theta )\right)}{2}$$

Therefore, as long as the input control variable of the system is obtained respectively when the motor is running at a uniform speed in the positive or negative directions, and their average values are obtained and then fed into the system, the cogging torque disturbance can be eliminated. Of course, there will still be some errors in this method for the system can barely maintain strict uniform speed, as well as other disturbance exists in the system.

### 3.2. Specific Design for ILC

It can be seen from the above analysis that in order to obtain feedforward control variable $u_{+}$ and $u_{-}$, it is necessary to make the motor run at a constant speed. In our experiment, we set the reference speed $\omega_{d}=10^{\circ }/s$.

Due to the limited memory of the processor, we set the sampling interval to 1°. The cogging torque is repeated once per circle, and the number of iterations is increased by one when the motor angle jumps from 360° to 0°. When the motor turns the integer angle value, the corresponding control variable is stored. For any position, the control variable corresponding to the last iteration is obtained by linear interpolation, that is:

(9) $$u_{k-1}(\theta )=\left(u_{k-1}\left([\theta ]+1\right)-u_{k-1}\left(\left[\theta \right]\right)\right)\cdot \left(\theta -[\theta ]\right)+u_{k-1}\left(\left[\theta \right]\right)$$

where [θ] represents the downward rounding of angle value, and $u_{k-1}\left(\left[\theta \right]\right)$ represents the corresponding control variable at the angle θ downward rounding of the ${\left(k-1\right)}^{th}$ iteration.

Since the disturbance torque in the actual system is not strictly repeated with the number of iterations, that is to say, $d_{k}$ and $d_{k+1}$ in Equation (5) are only approximately equal. In this case, the system error will continue to accumulate and even lead to system divergence. To solve this problem, we need to add a forgetting factor on the basis of Equation (2):

(10) $$u_{k}(\theta )=\left(1-\alpha \right)u_{k-1}(\theta )+G_{P}e_{k}(\theta )+G_{I}\int e_{k}(\theta )dt$$

where, α is the forgetting factor.

It can be concluded that the $k^{th}$ iteration error at this time is:

(11) $$e_{k}=\left(1-\alpha \right)e_{k-1}-F\left(e_{k}\right)+\alpha \omega_{d}+\left(1-\alpha \right)d_{k-1}-d_{k}$$

The above equation indicates that the introduction of forgetting factor can weaken the cumulative error caused by aperiodic disturbance.

**Figure 2:**
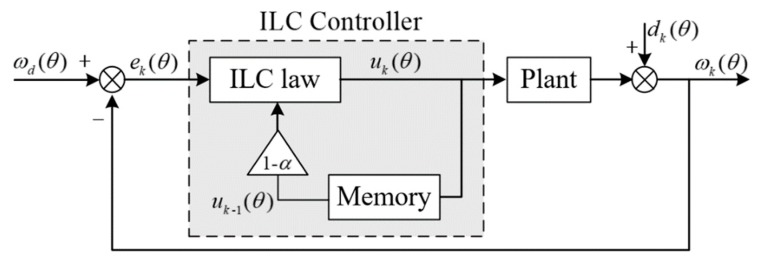
Block diagram of improved ILC.

The improved system block diagram is shown in Figure 2:

### 3.3. ILC Experiment

The ILC designed above is tested in our system. See Table 1 for the specific parameters of the PMSM driving the test turntable.

The main control chip of the platform is DSP (TMS320F28335) (Texas Instruments, Dallas TX, USA), and its working frequency is 150 MHz. Another CPLD (EPM570F100I5N) (Intel, Santa Clara, CA, USA) is used as the auxiliary control chip. The driver is DRV8312, powered by a 15 V linear regulated power supply. The mechanical angle of the motor is measured by a 12-bit magnetic encoder. A 14-bit micromechanical gyroscope is used to measure the speed of the turntable. Its noise amplitude is $\pm 0.25^{\circ }/s$, and the maximum position error obtained by static noise integration is $0.003724^{\circ }$. The phase currents are converted into voltages by 3 0.01Ω/0.25W/0.5% high precision power resistors, and then output to the input of a 16-bit ADC (AD7606) (Analog Devices, Norwood, MA, USA) through linear amplification circuit.

The current loop operates at 8 kHz and the speed loop operates at 1 kHz.

**Figure 3:**
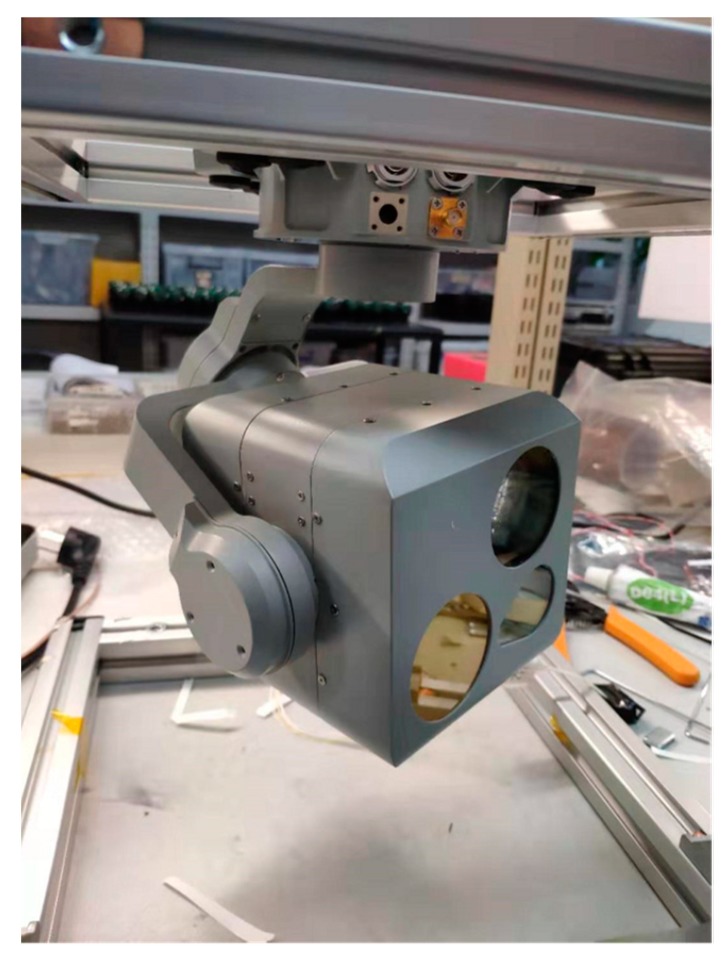
Experimental platform.

See Figure 3 for the experimental platform.

**Figure 4:**
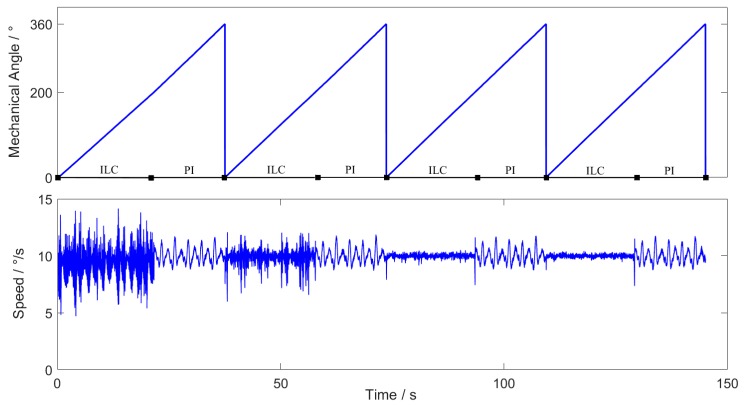
Experimental results of ILC.

In order to observe the effectiveness of the ILC in the experimental system more intuitively, the ILC is used at the mechanical angle $0^{\circ }\sim 200^{\circ }$, and the PI controller is used at the remaining positions. The reference speed is $10^{\circ }/s$. The experimental results are as shown in Figure 4: 

The controller parameters are as shown in Table 2.

According to the above experimental results, ILC achieves good effect in the third iteration. Because of the cogging disturbance, the speed fluctuation of PI controller is large after the system closed-loop, while that of ILC is much smaller than PI controller.

**Figure 5:**
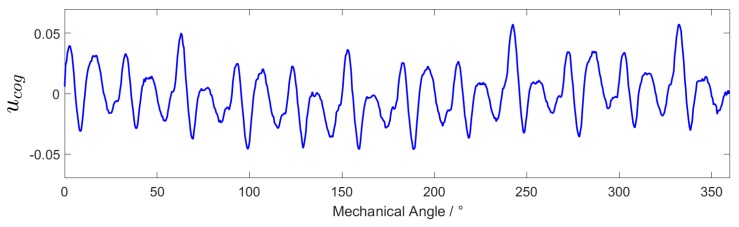
Variation of $u_{cog}$ with the mechanical angle θ.

Set the reference speed to $-10^{\circ }/s$, and set the range of action of ILC to all positions. It can be seen from the analysis in Section 3.1 that the algebraic average of the control variable in the two experiments after the system speed is stable is the control variable corresponding to the cogging torque. Denote the control variable as:

(12) $$u_{cog}=\frac{u_{+ILC}+u_{-ILC}}{2}$$

where $u_{+ILC},\;u_{-ILC}$ represent the control variables after the system speed is stable when the speed is positive and negative respectively. At the $1^{\circ }$ sampling interval, $u_{cog}$ is an array of size 360. The variation of $u_{cog}$ with the mechanical angle θ is shown in Figure 5:

### 3.4. Control Variable Feedforward to Restrain Cogging Effect

Because the cogging torque is almost independent of the working environment of the system, it is only related to the manufacturing process. We feed the control variable corresponding to the cogging effect in the experiment of Section 3.3 into the system, which can suppress the cogging effect offline. 

In the process of motor operation, read the value of mechanical angle θ in real time, and find the adjacent control variables $u_{cog}\left(\left[\theta \right]\right)$ and $u_{cog}\left(\left[\theta +1\right]\right)$ from $u_{cog}$ array. Where $u_{cog}\left(\left[\theta \right]\right)$ represents the corresponding value of the array $u_{cog}$ stored in the memory after the mechanical angle θ is rounded down. The $u_{cog}(\theta )$ obtained by linear fitting is the control variable of the final feedforward system, that is:

(13) $$u_{cog}(\theta )=\left(u_{cog}\left(\left[\theta +1\right]\right)-u_{cog}\left(\left[\theta \right]\right)\right)\cdot \left(\theta -[\theta ]\right)+u_{cog}\left(\left[\theta \right]\right)$$

**Figure 6:**
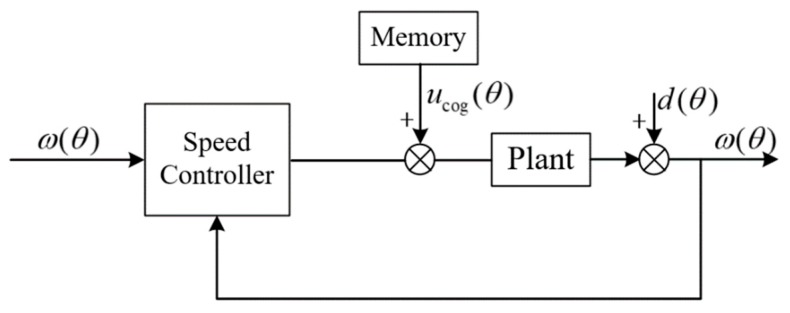
System diagram after ILC feedforward.

The system block diagram is shown in Figure 6:

## 4. Design of Sliding Mode Controller

The design of sliding mode controller is divided into two steps: 

(1) Choose the appropriate sliding surface;

(2) Design the approach law. 

The sliding surface is a hyperplane of state variables. State variables defined on the sliding surface should be equal to 0 or converge to 0 in a finite time. The design of approach law should ensure that the system can slide to the sliding surface in a finite time regardless of the working state, that is to say, the reachability condition of sliding mode is satisfied.

### 4.1. Traditional Sliding Mode Control (SMC)

The traditional sliding surface is generally taken as:

(14) $$s=\omega_{d}-\omega \;=0$$

The traditional constant reaching law is:

(15) $$\dot{s}=-k\mathrm{sgn}\left(s\right)$$

where sgn(⋅) is the sign function.

When the system has no external disturbance and measurement error, the value of k can be very small. However, the operation of PMSM is affected by the cogging torque and the change of motor parameters, which makes the external disturbance inevitable. Similarly, sensor noise also exists in the system.

Let the speed of the motor measured by the sensor be $\omega_{m}=\omega +\delta$, where ω is the actual speed and δ is the measurement error. The disturbance torque of the system including load disturbance, cogging effect and shafting friction is $T_{d}$. From the mathematical model Equation (1) of PMSM, we can obtain:

(16) $$\dot{\omega }={\dot{\omega }}_{m}-\dot{\delta }=\frac{K_{t}}{J}i_{q}-\frac{B}{J}\omega -\frac{T_{d}}{J}\;$$

In the design process of the sliding mode controller, the speed measured by the sensor is used, and the external disturbance is ignored. That is to say, ${\dot{\omega }}_{m}=\frac{K_{t}}{J}i_{q}-\frac{B}{J}\omega_{m}$. According to Equation (15), the output control variable of the speed loop sliding mode controller designed according to this assumption is obtained from the following equation:

(17) $${\dot{\omega }}_{d}-\frac{K_{t}}{J}i_{q}^{*}+\frac{B}{J}\omega_{m}=-k\mathrm{sgn}(s)$$

That is:

(18) $$i_{q}^{*}=\frac{J}{K_{t}}\left[{\dot{\omega }}_{d}+\frac{B}{J}\omega_{m}+k\mathrm{sgn}(s)\right]$$

According to Equation (14):

(19) $$\dot{s}={\dot{\omega }}_{d}-\dot{\omega }\;$$

Combining Equations (16), (18), (19) and considering that the current loop can track the given value stably, we can obtain:

(20) $$\begin{array}{ll} \dot{s} & ={\dot{\omega }}_{d}-\frac{K_{t}}{J}i_{q}^{*}+\frac{B}{J}\omega +\frac{T_{d}}{J}\\ & ={\dot{\omega }}_{d}-\frac{K_{t}}{J}i_{q}^{*}+\frac{B}{J}\omega_{m}+\frac{T_{d}}{J}-\frac{B}{J}\delta \\ & =-k\mathrm{sgn}\left(s\right)+\frac{T_{d}}{J}-\frac{B}{J}\delta \end{array}$$

In order to make the sliding mode controller satisfy the reachability condition, combining the above equations, we can obtain:

(21) $$\left\{ \begin{array}{l} -k+\frac{T_{d}}{J}-\frac{B}{J}\delta <0\;,\;when\;s>0\\ k+\frac{T_{d}}{J}-\frac{B}{J}\delta >0\;,\;when\;s<0 \end{array}\right.$$

That is,

(22) $$k>\left|\frac{T_{d}}{J}-\frac{B}{J}\delta \right|$$

When the external disturbance $T_{d}$ is large, k must take a value large enough to satisfy Equation (22). However, the increase of k value makes the state of the system cross the sliding surface more frequently, resulting in severe chattering [16,23].

### 4.2. Selection of Sliding Surface

In order to eliminate the steady-state error and weaken the chattering of the system, the integral sliding surface is selected [24]:

(23) $$s=e_{\omega }+c\int e_{\omega }dt$$

where $e_{\omega }=\omega_{d}-\omega$, and c is the integral coefficient.

### 4.3. SMC of New Approach Law

The sliding mode controller designed by the traditional constant speed approach law cannot deal with the contradiction between fast approach and chattering reduction [25]. For this reason, the following new approach law is designed:

(24) $$\dot{s}=\;-fe\left(s\right)\cdot s\mathrm{gn}\left(s\right)$$

(25) $$fe\left(s\right)=-\frac{k}{1+e^{-\beta \left(\left|s\right|-\alpha \right)}}$$

where k , α , β are constants greater than 0.

When the system state is far away from the sliding surface, i.e., s is large (let s>0), $-\frac{k}{1+e^{-\beta \left(\left|s\right|-\alpha \right)}}\;\cdot \mathrm{sgn}\left(s\right)\approx -k$, it tends to the sliding surface at a faster speed. When the system state is close to the sliding surface, i.e., s is smaller, $-\frac{k}{1+e^{-\beta \left(\left|s\right|-\alpha \right)}}\;\cdot \mathrm{sgn}\left(s\right)\approx -\frac{k}{1+e^{\alpha \beta }}$ is a small value. Compared with the constant speed approach law, this new approach law can make the system tend to the sliding surface at a very fast speed when the system state is far away from the sliding surface (desired state), and when the system state is close to the sliding surface, the coefficient of the signal function sgn(s) is very small, and the influence of the system state passing through the sliding surface frequently on the output of the controller is very small, which can effectively reduce the system chattering. At the same time, the coefficients α and β can adjust the speed of system state towards sliding surface when s takes different values, so as to better adapt to various practical systems. The coefficient k determines the maximum approach speed.

Combining Equations (14), (23), (24), the output of the new SMC controller proposed in this paper is as follows:

(26) $$i_{q}^{*}=\frac{J}{K_{t}}\left[{\dot{\omega }}_{d}+\frac{B}{J}\omega_{m}+ce_{\omega }+fe(s)\cdot \mathrm{sgn}(s)\right]$$

### 4.4. Stability Analysis

Define the Lyapunov function:

(27) $$V=\frac{1}{2}s^{2}$$

Then,

(28) $$\dot{V}=s\dot{s}$$

Substituting Equation (24) into Equation (28) yields:

(29) $$\dot{V}=s\left[-fe(s)\cdot \mathrm{sgn}\left(s\right)\right]$$

That is,

(30) $$\dot{V}=\left\{ \begin{array}{l} -s\cdot fe\left(s\right),\;\mathrm{when}\;s>0\\ s\cdot fe\left(s\right),\;\mathrm{when}\;s\leq 0 \end{array}\right.$$

From Equation (25), it can be seen that for any s, fe(s)≥0, so for any s, there is:

(31) $$\dot{V}\leq 0$$

According to the Lyapunov stability theory, the system with the proposed sliding mode controller is stable, and the sliding mode state variable, i.e., the speed tracking error $e_{\omega }$, can converge to 0 in a finite time.

### 4.5. Experimental Results of SMC with New Approach Law

In order to verify the effectiveness of the proposed sliding mode controller, the traditional SMC and the new SMC are used in the speed loop based on the off-line reduction of cogging torque in Section 3, and the experimental results are compared.

In order to ensure the validity of the experimental results, the initial position of the motor in each experiment is the same, the current loop controller is the same, and all the controller parameters have been adjusted to the optimal.

The experimental conditions are: uniform tracking with speed of $10^{\circ }/s$, sine wave tracking with amplitude of $10^{\circ }/s$ and frequency of 10 Hz, and triangular wave tracking with amplitude of $10^{\circ }/s$ and frequency of 1Hz.

**Figure 7:**
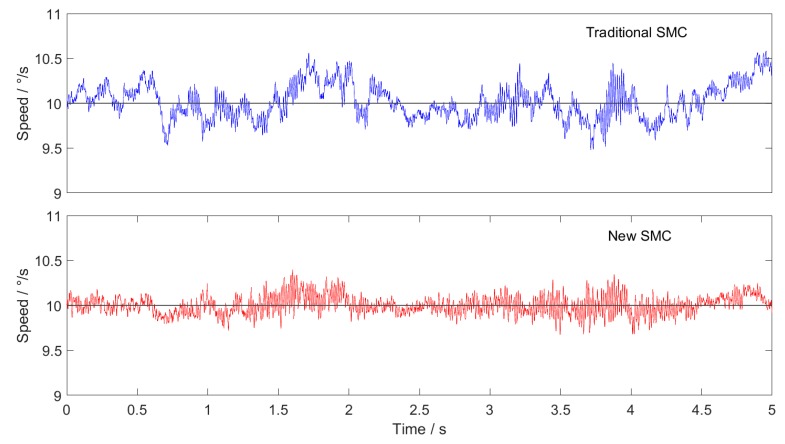
Tracking curve of uniform speed.

**Figure 8:**
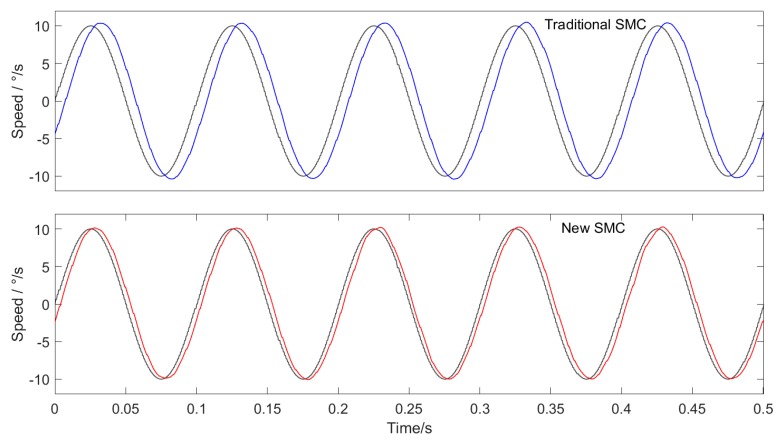
Tracking curve of sine wave.

**Figure 9:**
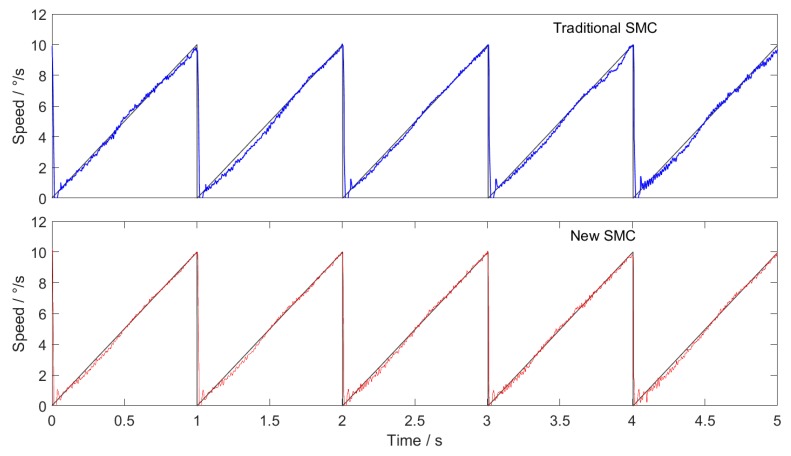
Tracking curve of triangular wave.

The nominal model of the turntable drive motor is $\dot{\omega }=18000i_{q}-10\omega$. The controller parameters are shown in the following Table 3:

The experimental results are shown in the figures below. Among them, Figure 7 shows uniform tracking, Figure 8 shows sine wave tracking and Figure 9 shows triangular wave tracking. The black line is the given speed, and the blue line and red line are the actual speeds.

The experimental results show that the new SMC + ILC feedforward method proposed in this paper has faster response speed and higher tracking accuracy than the traditional SMC + ILC feedforward method. The superiority of SMC based on the new approach law is proved.

## 5. Design of Compound Controller

### 5.1. Design of Extended State Observer (ESO)

It is known from Equation (22) that the speed of sliding mode controller moving towards the sliding mode surface due to external disturbance of the system cannot be too small, otherwise the reachability condition will not be satisfied. On the other hand, too fast an approach speed makes the system state frequently cross the sliding surface, resulting in system chattering. Although a new approach law is designed to improve the performance of the system, it cannot meet the requirements of high-precision servo system. Especially when the system works in the environment with external disturbance, such as the airborne inertial stabilization platform working in the air, it is affected by the aircraft swing, vibration and various air flow disturbances.

In order to improve the disturbance rejection ability of the system, an extended state observer is designed to observe the external disturbance. The observed disturbance is used to optimize the sliding mode controller.

From the dynamic equation of PMSM in Equation (1), we can obtain:

(32) $$\begin{array}{ll} \dot{\omega } & =\frac{K_{t}}{J}i_{q}-\frac{B}{J}\omega -\frac{T_{d}}{J}\\ & =\frac{K_{t}}{J}i_{q}^{*}-\frac{B}{J}\omega -\frac{T_{d}}{J}-\frac{K_{t}}{J}\left(i_{q}^{*}-i_{q}\right)\\ & =\frac{K_{t}}{J}i_{q}^{*}+d\left(t\right)\; \end{array}$$

Among them, $i_{q}^{*}$ is the reference value of current loop, i.e., the output control variable of SMC; $i_{q}$ is the actual value of q-axis current; $d\left(t\right)=-\frac{B}{J}\omega -\frac{T_{d}}{J}-\frac{K_{t}}{J}\left(i_{q}^{*}-i_{q}\right)$ is the total disturbance of the system, including shafting friction, cogging moment, load torque, q-axis current tracking error, etc.

Taking the PMSM mechanical speed ω and the total disturbance d(t) as the state variables and defining $x_{1}=\omega ,\;x_{2}=d\left(t\right)$, Equation (32) can be written in the form of the following state equation:

(33) $$\left\{ \begin{array}{l} {\dot{x}}_{1}=x_{2}+\frac{K_{t}}{J}i_{q}^{*}\\ {\dot{x}}_{2}=c\left(t\right) \end{array}\right.$$

where c(t) is the change rate of d(t).

In reference [26,27], the properties and design methods of extended state observer are analyzed in detail. According to this, the following linear ESO for system (20) is designed:

(34) $$\left\{ \begin{array}{l} {\dot{z}}_{1}=z_{2}-2p\left(z_{1}-x_{1}\right)+b_{0}i_{q}^{*}\\ {\dot{z}}_{2}=-p^{2}\left(z_{1}-x_{1}\right) \end{array}\right.$$

where *p* is the controller coefficient, and p>0, $z_{1}={\hat{x}}_{1}$ is the estimated value of PMSM mechanical speed, and $z_{2}=\hat{d}$ is the estimated value of disturbance, $b_{0}=\frac{K_{t}}{J}$.

### 5.2. Design of SMC + ESO

The output control variable of the sliding mode controller according to Equations (23), (24), (32) is:

(35) $$i_{qo}^{*}=\frac{J}{K_{t}}\left[{\dot{\omega }}_{d}+ce_{\omega }+fe\left(s\right)\cdot \mathrm{sgn}\left(s\right)\right]$$

The ESO designed in Section 4.1 is applied to our experimental system, and the total disturbance observed by Equation (34) is compensated into the sliding mode controller in order to obtain a new SMC + ESO composite controller. The output of the composite controller is given by the following equation:

(36) $$i_{qo}^{*}=\frac{J}{K_{t}}\left[{\dot{\omega }}_{d}+ce_{\omega }+fe\left(s\right)\cdot \mathrm{sgn}\left(s\right)-z_{2}\right]$$

where $s=e_{\omega }+c\int e_{\omega }dt$ is the sliding surface and $z_{2}=\hat{d}$ is the disturbance observed by ESO.

The input of SMC + ESO composite controller is the error $e_{\omega }$ of given speed $\omega_{r}$ and actual speed ω, as well as the disturbance estimation value $z_{2}$, and the output control variable $i_{qo}^{*}$ is the reference input of the current loop.

The parameter p of ESO determines the bandwidth of the observer. But when the value of p is too large, the observation noise will increase. So the selection of p should be balanced between the bandwidth and the noise. The larger the parameter c of the sliding mode controller is, the faster the system state will reach the sliding mode sliding surface, but at the same time the larger the overshoot of the system output will be. The value of k determines the maximum speed that the system state tends to the sliding surface. α and β are used to adjust the speed that the system state tends to the sliding surface when it deviates from the sliding mode surface to different degrees. After repeated experimental debugging, a group of satisfactory controller parameters are obtained, as shown in Table 4.

Reference [28] analyzes in detail how to make the observer achieve good results under the condition of ensuring the stability of the observer.

The off-line feed-forward method of ILC may have residual error in restraining cogging torque, but the experimental results in Section 3.3 show that the residual error is very small.

According to the stability analysis in Section 4.4, the system can keep stable when ESO is stable and ILC feedforward has a little error.

**Figure 10:**
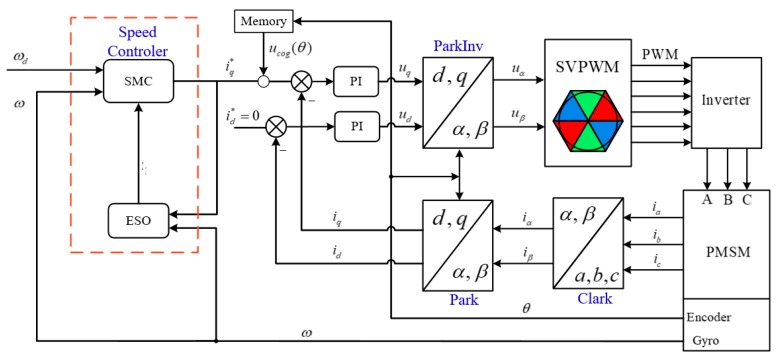
Control block diagram of PMSM based on compound controller.

The PMSM control block diagram based on speed loop SMC + ESO + ILC is shown in Figure 10.

### 5.3. Design of Comparative Experiment

The traditional PI + DOB controller of the speed loop is designed on the experimental platform, and compared with the ILC + SMC + ESO method.

**Figure 11:**
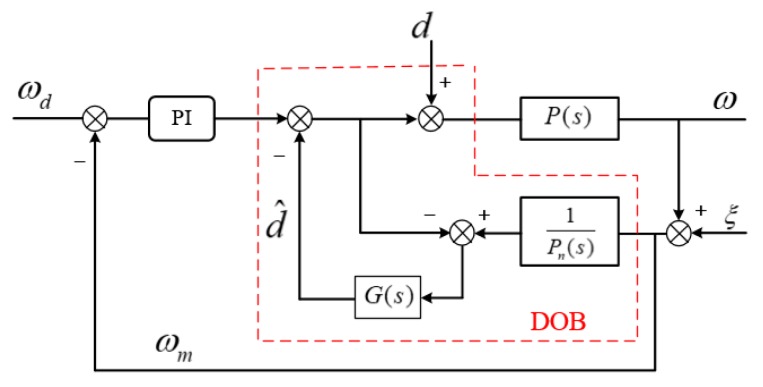
Block diagram of proportional integral (PI) + disturbance observer (DOB).

The structure diagram of PI + DOB is shown in Figure 11, where P(s) is the actual model from current $I_{q}$ to output speed ω, $P_{n}(s)$ is its nominal model, d is the external disturbance, which is observed by DOB.

### 5.4. Controller Parameters

The key of DOB is the design of low-pass filter g (s). G(s) affects the effect of noise observation and the stability of the system. The wider the G(s) bandwidth is, the more frequency components of the external disturbance d are observed, and the phase lag of $\hat{d}$ is smaller. Thus the closed-loop system has a stronger ability to suppress external disturbances. However, according to the small gain theorem, the stability of the system can be guaranteed only when ${\left\|\Delta (s)G(s)\right\|}_{\infty }\leq 1$. Where Δ(s) is the uncertainty of the system. Therefore, The G(s) bandwidth should not be too wide, otherwise the system will be unstable.

The PI controller is adjusted to ensure that the phase margin of the system is at least 30 to maximize the system bandwidth.

The optimal controller parameters can be obtained as shown in the Table 4.

## 6. Results and Discussion

**Figure 12:**
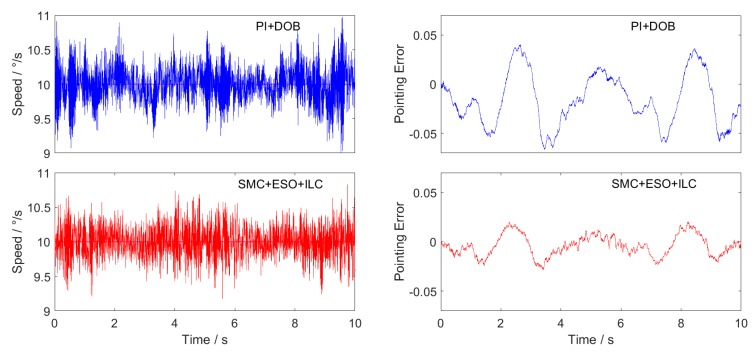
Uniform speed tracking curve and position pointing error.

**Figure 13:**
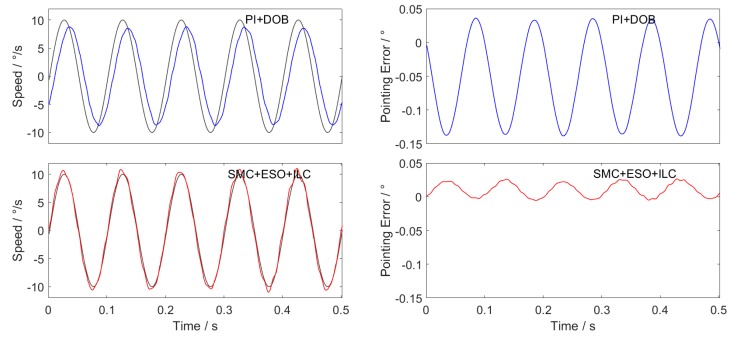
Sine wave tracking curve and position pointing error.

**Figure 14:**
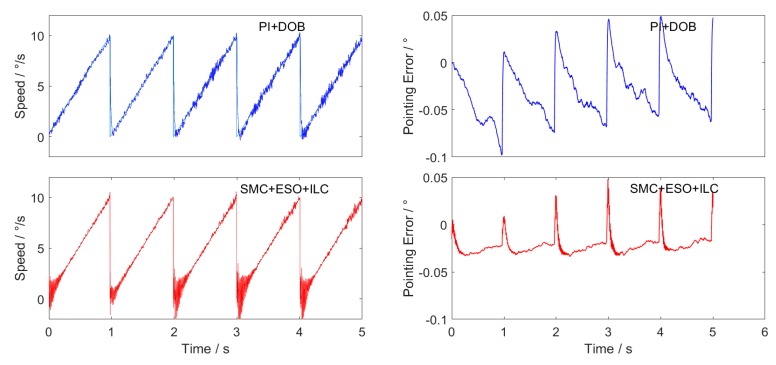
Triangular wave tracking curve and position pointing error.

The performances of the two control schemes are compared in the speed $10^{\circ }/s$ uniform tracking, amplitude $10^{\circ }/s$ frequency 10Hz sine wave tracking and amplitude $10^{\circ }/s$ frequency 1 Hz triangular wave tracking. The experiment is carried out on the platform shown in Figure 3. The hardware conditions of different groups of experiments are the same, and the current loop controller and its parameters as well as the driver scheme are also identical. Repeated experiments were carried out in multiple groups, and the difference between the experimental results was small. Typical result curves are shown in Figure 12, Figure 13 and Figure 14.

In this paper, the main function of PMSM is the stabilization control of the Los of the inertial stabilization platform, and the pointing error of the position is the final consideration standard.

The pointing error of the position is obtained by integrating the velocity error with the time, that is:

(37) $$\Delta \theta (t)=\int \left(\omega_{d}-\omega \right)dt$$

The sampling rate of the experimental data in this paper is 1kHz. For discretization of the above equation, we can obtain:

(38) $$\Delta \theta =\sum \left(\omega_{d}-\omega \right)\cdot 0.001$$

Repeat each experiment 5 times, calculate the pointing error with Equation (38), and take the average value of theta calculated from 5 groups of velocity error data as the position pointing error. The final results are shown in Table 3, Table 4 and Table 5.

Three experimental curves are obtained. The figures on the left of each group are the speed tracking curves, and on the right are the position pointing error curves obtained according to Equation (38).

The maximum value, average value and RMS value of the final position pointing error absolute value are shown in Table 5, Table 6 and Table 7.

## 7. Conclusions

The new sliding mode approach law proposed in this paper can effectively resolve the contradiction between the approach speed of the traditional constant speed approach law and the weakening of system chattering. The experimental results show that in the experiments of constant speed tracking, sine wave tracking and triangular wave tracking, the control effect of the sliding mode controller based on the new reaching law with ILC feedforward is superior to that of the traditional sliding mode controller with ILC feedforward.

When the sliding mode controller of the new approach law is combined with the extended state observer and ILC feedforward control scheme, the RMS value of the position pointing error is only 0.0068 ° in the constant speed-tracking experiment, which fully meets the accuracy requirements of the inertial stability platform, and the accuracy is more than two times higher that of the traditional PI + DOB control method. In the sine wave tracking experiment, there is obvious phase lag in the traditional PI + DOB control scheme, which makes the speed have a large tracking error. While the SMC + ESO + ILC control scheme proposed in this paper greatly reduces the phase lag, and the average value of the position pointing error is only 0.0118°, which is 1/6 of the PI + DOB control scheme. In the triangular wave-tracking experiment, the control scheme of SMC + ESO + ILC has overshoot and chattering when the given speed suddenly changes, but its tracking effect is better than that of PI + DOB at the given speed “Climbing” stage, and the final position pointing error RMS value is about 1/3 of PI + DOB.

## Figures and Tables

**Table 1:** Parameters of permanent magnet synchronous motor (PMSM).

Description	Value
Peak torque	≥0.75N⋅M
Continuous	≥0.15N⋅M
Rated voltage	24 V
Peak current	13.8 A
Armature resistance	11.8 Ω
Armature inductance	28 mH
Speed (Max. no-load)	3600 r/min
Number of pole pairs	6
Initia	$0.5\mathrm{Kg}\cdot m^{2}\times 10^{-5}$

**Table 2:** Controller parameters of ILC.

Parameter	Value
α	0.05
$G_{P}$	0.06
$G_{I}$	0.003

**Table 3:** Controller parameters.

Controller	Parameters			
Traditional sliding mode control (SMC)	k			
	1200			
SMC with new approach law	c	k	α	β
	10	1600	3.5	0.8

**Table 4:** Controller parameters.

Controller	Parameters				
PI + DOB	$K_{p}$	$K_{i}$		G(s) Bandwidth	
	0.0103	0.06		15 Hz	
SMC + ESO	c	k	α	β	p
	10	4000	20	0.2	300

**Table 5:** Position pointing error of uniform speed tracking.

	PI + DOB	SMC + ESO + ILC
Max(|Δθ|)	0.0666	0.0286°
Mean(|Δθ|)	0.0282	0.0102°
Rms(|Δθ|)	0.0166	0.0068°

**Table 6:** Position pointing error of sine wave tracking.

	PI + DOB	SMC + ESO + ILC
Max(|Δθ|)	0.1384°	0.0286°
Mean(|Δθ|)	0.0644°	0.0118°
Rms(|Δθ|)	0.0460°	0.0088°

**Table 7:** Position pointing error of triangular wave tracking.

	PI + DOB	SMC + ESO + ILC
Max(|Δθ|)	0.0979°	0.0479°
Mean(|Δθ|)	0.0354°	0.0236°
Rms(|Δθ|)	0.0206°	0.0063°
